# The Impact of CBT on Sick Leave and Health

**DOI:** 10.1177/0193841X20976516

**Published:** 2020-12-21

**Authors:** Pathric Hägglund, Per Johansson, Lisa Laun

**Affiliations:** 1 Swedish National Audit Office and SOFI, Stockholm, Sweden; 2 151672 Uppsala University, Sweden; 3 Institute for Evaluation of Labour Market and Education Policy (IFAU), Uppsala, Sweden; 4 Tsinghua Univiversity, Beijing, China

**Keywords:** rehabilitation, sick leave, health, cognitive behavioral therapy

## Abstract

This article analyses the effect of cognitive behavioral therapy (CBT) for individuals with mild or moderate mental illness. We study the effects on sick leave, health care consumption, and drug prescriptions. We find that CBT improved health and prevented sick leave for individuals who were not on sick leave when treatment was initiated but had no effect for individuals who were on sick leave when the treatment was initiated.

Sickness and disability benefits’ expenditures are substantial in many countries. In 2007, the average Organization for Economic Cooperation and Development (OECD, 2010) country spent 1.9% of gross domestic product on sickness and disability benefits or about 10% of public social spending. This was almost 3 times as much as the average cost of unemployment benefits. One of the most common causes of work absence due to illness, what we denote as sick leave, is mental disorders. About 20% of the population in an average OECD (2012) country suffers from mental illness at any point in time and up to 50% experiences mental illness at some point during their life.

Mental disorders severely affect the quality of social and working lives of individuals, and the costs to society are large, not only the direct costs to the health care system but also indirect costs such as decreased productivity and public benefit payments. Tackling mental health problems is a key challenge for modern society. Mild or moderate mental illness is far more common than severe mental disorders, accounting for as much as three quarters of mental disorders (OECD, 2012). Despite this, the bulk of the research concerns the latter group.

In this article, we analyze the impact of cognitive behavioral therapy (CBT) for individuals with mild or moderate mental illness. We use a propensity score matching approach and exploit a government initiative, the medical rehabilitation guarantee (MRG), that led to a gradual expansion of the supply of CBT. This implies that individuals with similar potential to benefit from treatment were exposed to treatment to varying degrees across time and residence in the country. The analysis focuses on individuals aged 20–64 who had a registered health care visit with a mental illness diagnosis that could qualify for CBT within the MRG during 2010–2012 in the Skåne county council in the south of Sweden. The reasons for focusing on Skåne are the availability of detailed individual health data, which enables us to find nontreated individuals who are similar to the treated individuals in terms of observed characteristics, and of detailed information on certified CBT clinics.

We analyze the effects of CBT on sick leave and other health-related outcomes, such as health care consumption and drug prescriptions, for up to 2 years after treatment. To study the benefits of CBT at different stages of the sickness episode, the analysis is performed separately for individuals receiving treatments before entering sick leave and when on sick leave. The results suggest that CBT improved health and prevented sick leave for individuals who were not on sick leave at the start of treatment but had no effect for individuals who were on sick leave, although the estimates are not significantly different. We also provide rough calculations of the public finance implications up to 2 years after the initiation of treatment, suggesting that CBT was cost effective as a preventive measure.

Evidence of what types of treatment can be effective not only in improving health but also in facilitating employment for individuals with mental illness is scarce. This is particularly true for labor market outcomes, for example, sick leave. Earlier studies suggest that psychological treatments, in particular CBT, lead to symptom improvements for anxiety and depression (for a review, see SBU, 2004). However, the more recent review by Nieuwenhuijsen et al. (2008) concludes that there is no evidence that medication alone or enhanced primary care reduces sick leave among depressed workers and that there is no evidence for or against the effectiveness of psychological interventions in terms of sick leave. We add to this literature by showing that CBT can be an effective treatment to reduce sick leave.

According to OECD (2012), evidence suggests that the effectiveness of drug treatments for mental illness increases with illness severity, whereas psychotherapy may be more effective to treat milder mental disorders. OECD (2012) also recognizes that symptom improvements for mental illness do not necessarily translate into improved employment outcomes. Mild or moderate mental disorders may eventually turn into severe disorders if no treatment is provided. In terms of employment outcomes, individuals may also benefit more from treatment at an earlier stage of the sickness episode when the attachment to the workplace is still strong. The evidence from a literature review is that vocational rehabilitation can be effective if provided at the workplace (Johansson et al., 2011). Our results indicate that CBT may be more effective for individuals with a milder illness who were not on sick leave at the start of treatment.

This article proceeds as follows. The second section provides the background about the government initiative and the induced supply of CBT in the Skåne county council. The third section describes the data. The fourth section outlines the empirical strategy and estimation. The fifth section presents the results, and the sixth section provides an estimation of the implications for the public finances. The seventh section concludes this article.

## The MRG

In 2008, the Swedish government launched an MRG providing additional funding to the county councils for adopting evidence-based treatments of mental illness and back and shoulder pain. Mental disorders and musculoskeletal diseases each accounted for about 30% of the total sick leave costs in Sweden by the time the program was introduced, and the purpose was both to prevent sick leave and to promote return to work for individuals with these diagnoses. The treatments qualifying for additional funding include CBT for individuals with mental illness and multidisciplinary treatment for individuals with back and shoulder pain. Since the target groups differ, the treatments are not substitutes for each other, and the evaluation does not concern the relative merits of the two types. This article focuses only on the CBT. The diagnoses qualifying for CBT treatment within the MRG are listed in the Appendix (Table A1).

### CBT Within the MRG

The purpose of CBT is to affect thoughts, feelings, and behavior in a positive direction by combining behavioral and cognitive therapy. Individuals learn to recognize difficult situations and to identify and implement an acceptable response. The approaches to achieve this can differ, but strategies with exercises and home assignments are important components (Swedish Government and Swedish Association of Local Authorities and Regions, 2011).

The MRG for CBT specified a two-step process to initiate treatment. First, a medical evaluation and a structured psychological assessment should be performed by qualified personnel, resulting in a diagnosis giving a picture of the syndrome, the personality, and the functioning in relation to work. After an initial assessment, individuals with mild or moderate mental illness should be offered CBT individually or in groups. The number of treatment sessions is individualized, but a treatment sequence should in general contain 10–15 sessions. The personnel must include a qualified psychologist or psychotherapist with CBT competence or a nurse, social welfare officer, physiotherapist, or physician with supplementary education within CBT.

Table 1 presents the descriptive statistics of the treatments for the participants in the MRG in Skåne during the period under study. The median treatment period was 105 days, and the median number of visits during this period was 7 and 8. The majority of the treated did not reach the recommended 10–15 sessions. Also, 5%–10% dropped out after two sessions. For CBT patients not on sick leave (on sick leave) at the initiation of treatment, 57% (55) of the sessions were handled by a psychologist, 15% (18) by a social worker, and 15% (16) by a psychotherapist. Almost two thirds of the sessions involved CBT and one third involved cognitive therapy.

**Table 1.** Description of CBT Treatment Within the MRG.

Variables	CBT Patients	
	Not on Sick Leave	On Sick Leave
Statistics per treatment period		
Treatment period, days		
Mean (standard error)	124 (91)	126 (92)
Median	105	105
Number of visits		
Mean (standard error)	7.3 (3.6)	7.8 (3.8)
Median	7	8
Observations	11,114	2,537
Statistics per meeting		
Treatment category, percentage		
Physician	1	1
Nurse	1	2
Physiotherapist	3	2
Social worker	15	18
Psychologist	57	55
Psychotherapist	16	15
Other	7	7
Type of treatment, percentage		
Systematic psychological treatment, cognitive	33	34
Systematic psychological treatment, CBT	62	61
Group treatment from manual method	2	3
Rehabilitation according to rehabilitation plan	2	3
Observations	92,633	22,243

*Source*. Skåne county council care database*. Note.* The sample includes the participants in the MRG in Skåne during January 1, 2010, to June 30, 2011, or January 1–December 31, 2012. CBT = cognitive behavioral therapy; MRG = medical rehabilitation guarantee.

**Figure 1.**
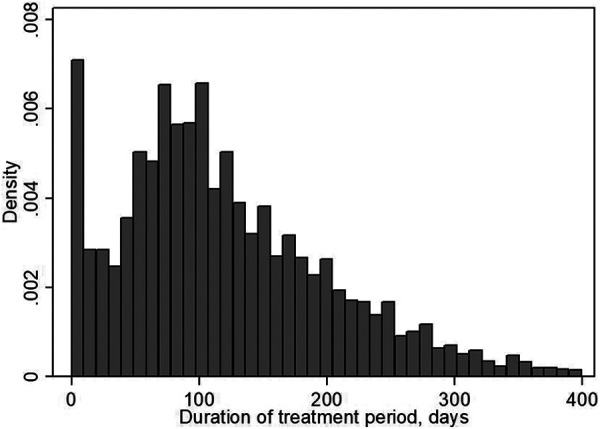
Distribution of the duration of the treatment period in days for the participants in the medical rehabilitation guarantee in Skåne.

Figure 1 presents the distribution of the duration of treatment within the MRG in Skåne. The figure shows that treatment typically lasted for a few months. Treatment was most intense at the beginning of the treatment period, with most meetings taking place during the first quarter.

### The Gradual Expansion of CBT in the Skåne County Council

**Figure A1.**
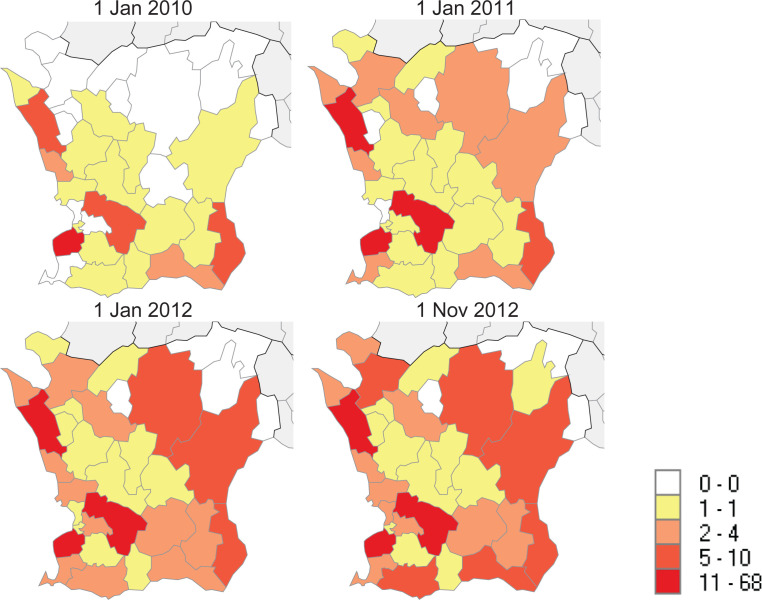
Number of contracted clinics for cognitive behavioral therapy within the medical rehabilitation guarantee in the Skåne municipalities.

**Figure A2.**
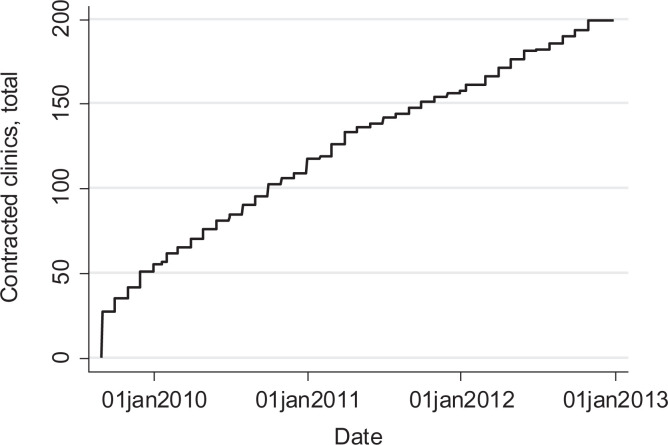
Total number of clinics with a contract to perform cognitive behavioral therapy within the medical rehabilitation guarantee in Skåne from September 1, 2009, to December 31, 2012.

Because of the lack of personnel with adequate qualifications, the number of clinics with a contract to provide CBT increased gradually in Skåne. Initially, clinics that already had the competence to provide CBT applied for the contracts. The county council supported education programs and other efforts to increase the number of certified personnel and thereby the supply of CBT treatments, which resulted in an expansion of certified clinics. Clinics could also hire qualified personnel and thereby fulfill the requirements to apply for a contract. The number of contracts varied across municipalities and increased steadily over time in all municipalities (see Figures A1 and A2 in the Appendix).

The assessment of whether the individual qualified for CBT within the MRG should be made at the primary care unit where the patient was listed. After that, the individual could choose among the contracted clinics within the entire Skåne county council. The remittance was made by the doctor at the primary care unit, and treatment should begin within 1 month after the remittance arrived at the contracted clinic. Since Skåne is one county council, individuals in Skåne have access to the same health care no matter in which municipality they live. Thus, the patients could choose a clinic in any part of Skåne. The supply of CBT in the own municipality was therefore not a restriction of receiving CBT treatment. The probability of receiving treatment is, however, likely to be correlated with the number of certified clinics in the area of residence. This may, for example, be due to the patients being more prone to participate in treatment if it can be provided nearby or to the doctor more frequently proposing treatment if there are certified clinics in the area.

**Figure A3.**
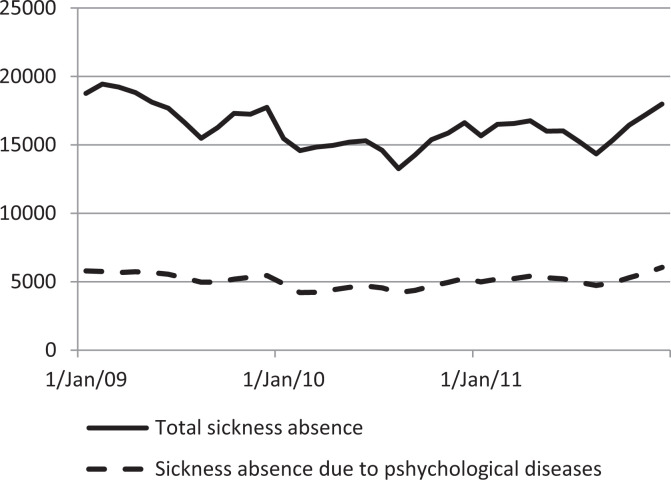
Total sick leave and sick leave due to psychological diseases in the Skåne county January 1, 2009, to December 1, 2011, number of individuals at the beginning of each month.

We argue that the implementation of the MRG in Skåne and the gradual expansion of CBT can be seen as exogenous. First of all, the decision about the MRG was taken by the Swedish government and not the county council in Skåne. Hence, the reform was based on the health development in the country as a whole rather than the situation in Skåne. Figure A3 shows the sick leave prevalence in Skåne at the beginning of each month from January 1, 2009, to December 1, 2012. The figure displays both total sick leave and sick leave due to psychological diseases. From this figure, we can see that the overall sick leave is stable or even potentially decreasing over the period under study. Sick leave due to psychological diseases is very stable over the period. This suggests that the general expansion of CBT cannot be seen as a response to an increase in demand for these services.

Second of all, we find no indications of the expansion of certified CBT clinics at the local level being driven by the demand for CBT. For example, we have investigated the relationship at the municipality level between the number of health care visits with a CBT diagnosis as a share of the population during the first quarter of 2008, before the MRG, and the final number of contracted clinics at the end of 2012 (both as total contracts and in relation to the size of the population). There is no significant correlation between these variables, suggesting that the rate of expansion of CBT is not correlated with the initial degree of illness in the area. Furthermore, according to the data on type of treatment, there are very few registered health care visits with cognitive therapy or CBT in 2008, prior to the MRG. Finally, we do not find a change in the share of diagnosed health care visits with a mental illness diagnosis at the passing of the MRG. In sum, the evidence does not give rise to concerns that the expansion of CBT was demand driven.

## Data

We use data from the health care databases in the Skåne county council, which contain detailed individual-level information about all health care visits in the county from January 1, 2008, to August 31, 2013. Skåne is a council in the south of Sweden, which covers 33 of Sweden’s 290 municipalities and has a population of about 1.25 million individuals of about 9.5 million in all of Sweden. We have also added information about drug prescriptions from the National Board of Health and Welfare from January 1, 2008, to August 31, 2013. From the Social Insurance Agency, we have further collected information about all sickness and disability benefit spells from January 1, 2000, to August 31, 2013, along with a large set of individual characteristics such as age, education, marital status, employment status, earnings, and municipality of residence. In all, we have rich information on matters closely related to the individuals’ health and labor market position—factors that should be important for analyzing future sick leave, health care, and drug consumption.

The target population in this article is individuals aged between 20 and 64 who had a registered health care visit with a mental illness diagnosis that could qualify for CBT within the MRG between January 1, 2010, and June 30, 2011, or between January 1 and December 31, 2012.^1,2^ In total, 21% of the individuals in the target population for CBT received CBT within the MRG at some point during this period. That the shares are not higher may be due to a diagnosis not detailing a pati,nt’s suitability to receive treatment or to demand exceeding the supply of treatment. We follow the individuals up to 2 years after the start of treatment. As our outcome data end on August 31, 2013, individuals who begin to receive CBT between January 1, 2010m and June 30, 2011, can be followed for the entire 2-year period, whereas individuals who begin to receive CBT during 2012 can be followed between 8 and 20 months.

Table 2 presents the descriptive statistics for the target population by treatment status. The unit of observation is health care visits with a mental illness diagnosis. The first health care visit of a CBT sequence is defined as the individual treatment within the MRG, and the comparison group consists of all health care visits with a mental illness diagnosis for individuals who did not take part in the MRG (see Table A1 in the Appendix for a list of these diagnosis).

**Table 2.** Descriptive Statistics by Treatment Status for the Target Population (Before Treatment).

Variables	Cognitive Behavioral Therapy		
	Treated	Untreated	*t* Value
Male	0.30 (0.46)	0.34 (0.47)	−9.78
Age	39.69 (11.78)	41.91 (11.74)	−21.91
Foreign born	0.14 (0.34)	0.24 (0.43)	−34.79
College	0.50 (0.50)	0.34 (0.48)	36.33
On sick leave	0.18 (0.39)	0.26 (0.44)	−21.08
Unemployed	0.17 (0.38)	0.24 (0.43)	−20.47
Disability benefits	0.06 (0.25)	0.17 (0.39)	−51.86
Outpatient care visits since 2008			
Total	47.42 (48.35)	86.19 (126.10)	−77.10
Doctor’s visits	23.26 (21.96)	30.10 (29.71)	−35.05
Mental illness diagnosis	1.79 (3.51)	24.74 (93.64)	−100.24
Pain-related diagnosis	0.66 (2.52)	0.98 (3.00)	−14.22
Value of drug prescriptions since 2008	17,731 (141,279)	26,089 (101,465)	−7.01
Number of sick leave days last 3 years	56.61 (143.64)	109.61 (221.82)	−40.71
Observations	14,683	169,905	

*Note.* Standard deviations are given in parentheses. *t* value for the difference in means, absolute values above 1.96 indicate a statistically significant difference at the 5% level.

Table 2 shows that selection into CBT was fairly systematic, targeting healthier individuals with higher education and a better labor market situation than the population in general. The number of health care visits is much lower for treated individuals, as well as the value of previous drug prescriptions. Treated individuals are also unemployed and on sick leave to a lower extent than those not selected. Foreign born are underrepresented, and college educated are overrepresented among the treated CBT patients compared to those not treated.

We study three different outcome variables. The first is the number of sick leave days. Since data only include sickness insurance payments (and not sick pay from the employer), only sick leave episodes longer than 14 days are analyzed. As the sickness and disability insurance are closely tied together in Sweden, we also include periods of disability benefits into the sick leave outcome. This means that we do not separate between temporary and more permanent sick leave. The second outcome variable is the number of health care visits. This is directly related to treatment during the treatment period but is also an important health indicator after treatment has ended. The third outcome variable is the number of drug prescriptions. This may be an alternative treatment method during the treatment period but is also an interesting health indicator in the long run.

## Empirical Strategy

Our interest is in estimating the average treatment effect for those receiving CBT. To this end, we use a matching approach that exploits the randomness in the probability of receiving treatment due to the gradual expansion of CBT across the Skåne county council.^3^ The basic idea with matching is to recreate the randomized experiment ex post, based on pretreatment covariates. Given the design, the average treatment effect is simply estimated by taking the difference in estimated means between the treated and matched controls.

The central assumption in matching, known as the unconfoundedness or conditional independence assumption, is that we can control for all covariates that jointly affect treatment and outcomes if not treated. This is in general a strong assumption. The weakness with the strategy is that we do not know whether the design also balances unobserved covariates as is the case in the ideal randomized experiment. A large literature (see, e.g., Imbens and Rubin, 2015, for an overview) has dealt with this problem and suggested sensitivity analyses to this form of hidden bias. Some of these approaches suggest the possibility of using historical outcomes, not using the matching algorithm in a sensitivity analysis (cf. Rosenbaum, 1999). The idea is that there should not be any difference in these outcomes between treated and controls before treatment is given. Based on an additional variable, a more formal test is suggested in de Luna and Johansson (2014). Sensitivity Analysis section provides a discussion of the sensitivity of confounding in the design described below.

We have available a rich set of covariates measuring the patient’s current health status (e.g., previous health care visits, previous drug prescriptions, previous sick leave, and diagnosis), labor market status, socioeconomic, and demographic variables. This fact makes it more plausible that the unconfoundedness assumption holds than with a more limited set of covariates.

The second assumption is that it should be a random, or probabilistic, assignment conditional on the individual’s covariates. This is known as the common support or overlap condition. These two assumptions guarantee that we can find comparable individuals to the ones treated given the covariates.^4^

In all analysis, and perhaps especially so with rich data, it is important to discuss why we can find comparable individuals to the treated, or in other words, why one “twin” is treated while another is not. In our setting, all individuals belong to the same county council, which is responsible for the health care provision for the inhabitants. This means that all individuals have the right to obtain CBT but that the probability to be treated will depend on the supply in the local community. The supply in general will not depend on a single individual’s health. The expansion of CBT as a result of the MRG makes the assumption of random assignment conditional on covariates more plausible.

In the estimation, we use a one-to-one “nearest neighbor” propensity score matching estimator (see, e.g., Abadie & Imbens, 2006).^5^ This means that we find a nontreated “twin” to a treated individual by finding the closest nontreated individual, in terms of the predicted propensity score. We are not allowed to condition on future covariates or treatments. This means that untreated individuals at the time of matching could receive CBT treatment at a later point. The propensity of receiving CBT is estimated using logistic regressions. For each health care visit in which an individual begins to receive CBT within the MRG, we identify a health care visit for an untreated comparison group member with the same propensity to receive treatment.^6^ These matched health care visits are defined as the “start” of treatment for the treated and control groups. The analysis captures the effect of CBT within the MRG compared to ordinary treatment.

An important purpose of this article is to investigate whether treatment efficacy depends on the stage of the sickness episode. Therefore, all analyses are performed separately for individuals who were, and were not, on sick leave at the start of treatment. We also analyze dynamics by estimating the effects separately for each quarter since the start of treatment.

An important aspect with nonparametric estimators is that they have asymptotically nonignorable bias with many covariates (e.g., Abadie & Imbens, 2006). It is thus essential to work with as few covariates as possible. Given that there is no apparent and clear theory for which covariates should be considered as more important than others, the matching approach is implemented using a sequential design. The sequential design implies that we include variables as long as the mean absolute standardized value between the treated and controls of any covariate is larger than 0.25, which is the rule of thumb suggested in Imbens and Wooldridge (2009).^7^

Individuals with more than 10 previous health care visits with a mental illness diagnosis were eliminated from the beginning to increase comparability between the groups already before the matching. Furthermore, we restrict the controls to health care visits with a mental illness diagnosis in the same period as the treated (i.e., January 1, 2010, to June 30, 2011, or January 1, 2012, to December 31, 2012). The sequential design starts by adding the health variables, then the labor market status variables, and finally the socioeconomic and demographic variables. We wanted to avoid using previous sick leave as covariates to be able to use the lagged outcomes before estimation in a sensitivity analysis (there should not be an “effect” on these outcomes before treatment). For the individual not on sick leave receiving CBT, we did not need to include any lags of sick leave in order to obtain balance in the other covariates. For the individuals on sick leave, we had to include the two first lags of sick leave in order to obtain balance in the covariates. The models are fitted with a maximum of a second-order interaction term, although no interaction term was ultimately necessary to include.

**Figure A4.**
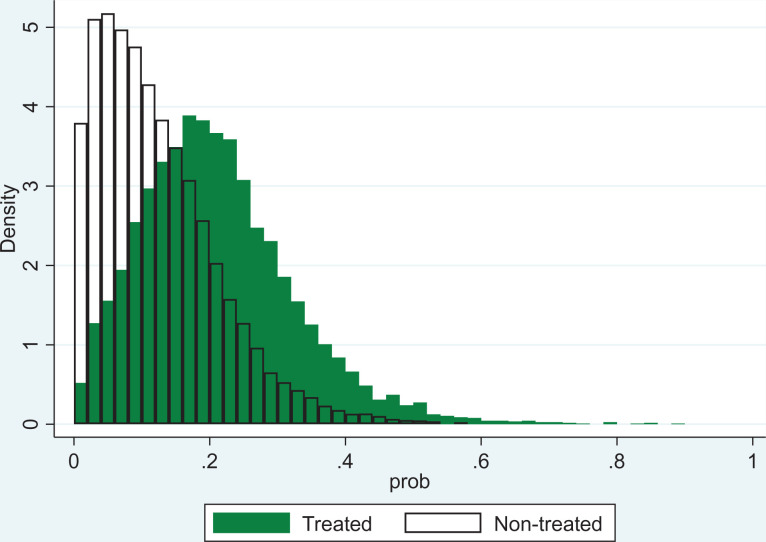
Distribution of estimated propensity scores for the treated and nontreated for the cognitive behavioral therapy sample before matching, not on sick leave.

Due to the sequential procedure, the estimated regression models differ across the two groups—those not on sick leave and those on sick leave at the time of treatment—which suggests that the selection to treatment may be different (for details, see Tables A2 and A3 in the Appendix). The propensity score of the treated and controls, both before and after matching, for those not on sick leave and on sick leave at the time of treatment is displayed in Figures A4a–[Fig fig7-0193841X20976516] [Fig fig8-0193841X20976516] A5b. The two distributions differ somewhat before matching, but there is an overlap in the population. After matching, the two distributions for both those not on sick leave and those on sick leave at the time of treatment have almost a perfect overlap.

Table 3 presents the descriptive statistics for the matched samples. After the matching, the characteristics of treated and untreated individuals are very similar, not only with respect to the variables included in the regression models but also with respect to the variables that were not included. Note, for example, that the history of sick leave for those not on sick leave and sick leave lagged 3 and 4 years for those on sick leave, which was never considered in the matching, is balanced across the matched samples.^8^ All in all, this provides support for the identification of the average treatment effects for those prescribed CBT.

**Table 3.** Descriptive Statistics by Treatment Status for the Matched Cognitive Behavioral Therapy Samples (Before Treatment).

Variables	Not on Sick Leave		On Sick Leave	
	Treated	Untreated	Treated	Untreated
Male	0.32 (0.47)	0.32 (0.47)	0.26 (0.44)	0.25 (0.43)
Married	0.33 (0.47)	0.33 (0.47)	0.44 (0.50)	0.42 (0.49)
Age	37.73 (11.41)	37.76 (11.41)	42.99 (10.58)	42.55 (10.38)
Foreign born	0.14 (0.34)	0.13 (0.34)	0.13 (0.33)	0.13 (0.34)
Earnings (SEK 1,000)	223.3 (165.5)	229.4 (310.2)	275.2 (118.5)	274.0 (131.0)
College	0.53 (0.50)	0.53 (0.50)	0.48 (0.50)	0.48 (0.50)
Unemployed	**0.17 (0.37)**	**0.16 (0.36)**	0.14 (0.34)	0.13 (0.33)
Number of prescriptions, since 2008	34.82 (46.04)	35.14 (45.45)	54.16 (72.01)	55.95 (79.25)
Specialist care visits, since 2008	12.98 (19.30)	12.61 (24.55)	20.01 (28.47)	19.20 (37.74)
Primary care visits, since 2008	26.53 (25.21)	26.54 (24.92)	36.68 (36.01)	35.51 (32.27)
Inpatient care days, since 2008	1.15 (5.62)	1.23 (6.67)	3.39 (15.29)	3.61 (14.91)
Doctor visits, since 2008	19.25 (16.54)	19.00 (17.77)	28.64 (24.47)	28.71 (32.98)
Total care visits, since 2008	39.13 (36.32)	38.78 (39.14)	56.00 (52.50)	53.95 (53.44)
Care visits, mental illness diagnosis, since 2008	1.28 (1.79)	1.28 (1.74)	1.91 (2.18)	2.00 (2.02)
Sick leave days, Quarter 1	1.87 (9.08)	1.65 (8.47)	46.07 (30.79)	46.31 (32.12)
Sick leave days, Quarter 2	1.90 (10.39)	1.74 (9.74)	18.60 (31.88)	20.33 (33.46)
Sick leave days, Quarter 3	1.94 (10.73)	1.69 (10.06)	12.41 (28.04)	13.29 (28.52)
Sick leave days, Quarter 4	1.87 (10.64)	1.91 (10.88)	10.42 (25.94)	10.80 (26.33)
Sick leave days, last 3 years	25.76 (88.82)	25.80 (94.39)	150.29 (208.26)	161.27 (223.13)
Observations	10,824	10,824	2,527	2,527

*Note*. Standard deviations are given in parentheses. Boldface values indicate statistically significant difference between treated and untreated at the 5% level.

Table 4 presents the descriptive statistics for treated and matched controls for the two groups during the period of CBT treatments. We determine the treatment period by the duration of CBT treatment within the MRG for the treated individual and assign the same duration counted from the start date of treatment to the matched, untreated individual. Thereby, we can compare the treatments during the period for which the treated individual participates in the MRG. No tests are presented, but with few exceptions, the differences between treated and untreated are strongly significant. Table 4 shows that the treated individuals receive substantially more health care visits in total during the treatment period. This is entirely due to an increased number of CBT visits by a median of seven to eight visits, compared to a median of one for the untreated.

**Table 4.** Description of Treatment for Matched Sample During Treatment Period of the Match.

Variables	Not on Sick Leave		On Sick Leave	
	Treated	Untreated	Treated	Untreated
Number of visits				
Mean (standard error)	12.21 (12.42)	6.07 (9.90)	18.23 (17.69)	10.72 (16.08)
Median	10	2	14	6
Number of CBT visits				
Mean (standard error)	7.04 (5.10)	1.82 (3.11)	8.58 (6.28)	3.01 (4.81)
Median	7	1	8	1
Number of visits per treatment category				
Physician	2.66 (4.49)	2.74 (4.26)	5.49 (6.66)	4.80 (7.39)
Nurse	1.13 (2.85)	1.12 (3.4)	2.02 (4.85)	1.96 (5.79)
Physiotherapist	1.03 (4.02)	0.42 (2.34)	2.17 (6.23)	0.98 (3.88)
Occupational therapist	0.07 (0.70)	0.06 (0.53)	0.18 (1.23)	0.13 (0.81)
Chiropractor/naprapath	0.06 (0.68)	0.02 (0.29)	0.07 (0.66)	0.02 (0.29)
Social worker	1.14 (3.17)	0.55 (1.92)	1.61 (3.91)	0.98 (2.72)
Psychologist	4.10 (4.93)	0.68 (2.23)	4.35 (5.39)	1.06 (3.14)
Psychotherapist	1.15 (3.29)	0.12 (0.95)	1.17 (3.39)	0.19 (1.44)
Other	0.85 (2.74)	0.32 (1.71)	1.11 (3.53)	0.51 (2.14)
Number of visits per type of treatment				
Systematic psychological treatment, cognitive	2.16 (4.06)	0.13 (0.90)	2.33 (4.25)	0.18 (1.11)
Systematic psychological treatment, CBT	3.88 (4.43)	0.27 (1.33)	4.15 (4.74)	0.32 (1.56)
Group treatment from manual method	0.14 (0.98)	0.01 (0.20)	0.26 (1.31)	0.02 (0.37)
Team rehabilitation	0.025 (0.55)	0.00 (0.19)	0.09 (1.08)	0.01 (0.35)
Rehabilitation according to rehabilitation plan	0.16 (1.21)	0.01 (0.32)	0.34 (2.20)	0.08 (1.10)
Other	0.45 (1.73)	0.39 (2.03)	0.76 (2.37)	0.70 (3.16)
Observations	10,824	10,824	2,527	2,527

*Note*. The treatment duration of the treated individual is assigned to the untreated match to define the “treatment period.” Note that the untreated individuals matched to the treated individuals at the initiation of treatment could receive CBT treatment within the medical rehabilitation guarantee at a later point. CBT = cognitive behavioral therapy.

Primarily, the increase in the number of health care visits for the treated individuals comes from the number of health care visits with a psychologist, psychotherapist, physiotherapist, or social worker. Treated individuals who were on sick leave at the initiation of treatment also meet a physician slightly more often than the untreated. In terms of the type of treatment, treated individuals primarily receive more health care visits with CBT or cognitive therapy compared to the untreated.

## Results

Table 5 presents the results (the average treatment effect of the treated estimates and their estimated standard errors^9^) on the effects of CBT for (1) the number of sick leave days (including days on disability benefits), (2) the number of health care visits, and (3) the number of drug prescriptions, during a follow-up period of up to 2 years after the initiation of treatment. The first panel in Table 5 presents the results for the entire period, whereas the two following panels present the results separately for the two subperiods, January 1, 2010, to June 30, 2011, and January 1, 2012, to December 31, 2012. Note that the follow-up period includes both the few months when the treatment group may have been receiving treatment and the subsequent months after the CBT treatment had ended. Thus, in the period when receiving the CBT treatment, Outcome (2) is a consequence of the treatment itself and Outcomes (1) and (3) may be a consequence of the treatment itself. In Table 6, we compare outcomes during and after treatment.

**Table 5.** Matching Impact Estimates of Cognitive Behavioral Therapy During a Follow-Up Period of Up To 2 Years.

Variables	Entire Period		2-Year Follow-Up		8–20 Months Follow-Up	
			January 1, 2010, to June 30, 2011		January 1, 2012, to December 31, 2012	
	Not on Sick Leave	On Sick Leave	Not on Sick Leave	On Sick Leave	Not on Sick Leave	On Sick Leave
Sick leave days	−5.6*** (1.0)	0.9 (5.8)	−6.6*** (1.7)	0.9 (10.0)	−4.7*** (1.0)	1.5 (6.1)
Mean of dep. var. in control group	22.7	199.0	29.3	234.4	16.7	167.6
Health care visits	1.7*** (0.4)	1.4 (1.0)	1.1* (0.6)	1.2 (1.7)	2.3*** (0.4)	1.7 (1.1)
Mean of dep. var. in control group	23.6	37.2	29.7	45.5	18.1	29.8
Drug prescriptions	−1.4*** (0.3)	−2.4*** (0.9)	−1.9*** (0.5)	−1.1 (1.6)	−1.0*** (0.3)	−3.1*** (1.0)
Mean of dep. var. in control group	12.2	21.2	16.9	27.7	8.0	15.5

*Note*. Standard errors are given in parentheses. Number of observations, entire period, not on sick leave: 17,561, on sick leave: 4,240. Two-year follow-up, not on sick leave: 9,360, on sick leave: 2,174; 8–20 months follow-up, not on sick leave: 9,820, on sick leave: 2,398.

** Statistical significance at the 5% level. ***Statistical significance at the 1% level.

**Table 6.** Matching Impact Estimates of Cognitive Behavioral Therapy During and After Treatment.

Variables	During Treatment		After Treatment	
	Not on Sick Leave	On Sick Leave	Not on Sick Leave	On Sick Leave
Sick leave days	−0.66** (0.34)	8.81*** (2.36)	−6.14*** (0.79)	−1.79 (1.70)
Health care visits	3.91*** (0.16)	4.04*** (0.50)	−3.19*** (0.28)	−3.96*** (0.80)
Drug prescriptions	−0.10 (0.10)	−0.51 (0.38)	−1.36*** (0.20)	−2.07*** (0.65)

*Note*. Standard errors are given in parentheses. Number of observations, not on sick leave: 17,561, on sick leave: 4,240. The treatment duration of the treated individual is assigned to the untreated match, in order to distinguish “during” and “after” treatment.

** Statistical significance at the 5% level. ***Statistical significance at the 1% level.

For those not already on sick leave at the start of CBT, sick leave was reduced by almost 6 days over the 2-year follow-up period or by about 25% compared to the mean in the control group. The number of health care visits increased by 1.7 visits, or by about 7%, which partly captures the CBT treatments. The number of drug prescriptions was reduced by 1.4 or by about 11% compared to the mean in the control group. When CBT was given to individuals already on sick leave, however, there was no significant effect on sick leave or health care visits, but there was a reduction in drug prescriptions. The results for the two subperiods are presented in the middle and last panel of Table 5. As the evaluation period for the second period is shorter, one can expect the effects, especially on sick leave, to be somewhat smaller in this subperiod. However, the results show no significant differences in the effect over time. Some estimates are slightly larger during the first period (as expected for sick leave), whereas others are larger in the second period.^10^

**Figure 2.**
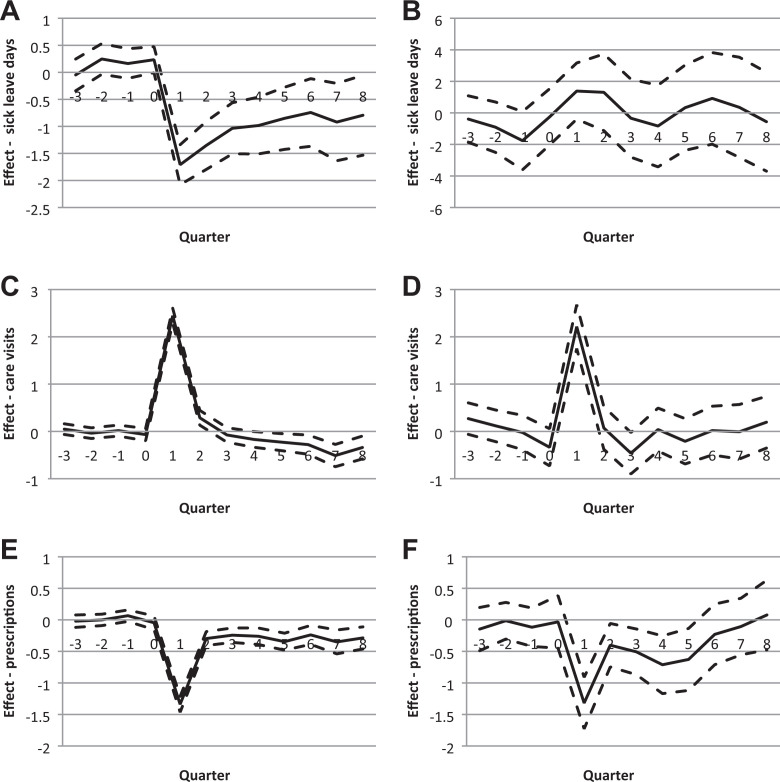
Matching estimates (mean solid line, dashes 95 confidence interval) of the effect of cognitive behavioral therapy on sick leave days, outpatient care visits with mental illness diagnosis, and the number of drug prescriptions, by sick leave status at the initiation of treatment. (A) Effect on sick leave days, not on sick leave. (B) Effect on sick leave days, on sick leave. (C) Effect on outpatient care visits with mental. (D) Effect on outpatient care visits with mental illness diagnosis, not on sick leave illness diagnosis, on sick leave. (E) Effect on drug prescriptions, not on sick leave. (F) Effect on drug prescriptions, on sick leave.

Figure 2 presents the dynamics of the results from four quarters before to eight quarters after initiation of treatment. The figure shows that there were no significant differences in the outcome variables between the treatment and the control group before treatment.

For individuals who were not on sick leave when treatment was initiated, Figure 2A shows that CBT immediately reduced the number of sick leave days following treatment by about 1.5 days. Even though the difference is decreasing over time, there is still a significant negative effect on sick leave by about 1 day eight quarters after the initiation of treatment. We know from Figure 1 that most treatment periods last for one to two quarters. For the same group of individuals, Figure 2C shows that the number of outpatient care visits with a mental illness diagnosis increased during the first and second quarter, which likely captures the more intensive CBT compared to alternative treatments. From three quarters onward, however, there is a significant decline of up to 0.5 visits for the treated compared to the controls, which is still significant eight quarters after the initiation of treatment. Figure 2E shows that drug prescriptions were also substantially reduced among CBT patients who were not on sick leave when treatment was initiated. Although the drop is most striking during the treatment period, this decrease is significant during the entire eight quarter follow-up period. Overall, the results suggest that CBT as a preventive treatment was successful in terms of reduced sick leave, reduced outpatient care visits in the long run, and reduced drug prescriptions.

For CBT given to individuals on sick leave, Figure 2B shows no significant effects on sick leave. Figure 2D shows that CBT increased the number of health care visits with a mental illness diagnosis in the short run but had no significant effect from the second quarter onward. As for the effect on the number of drug prescriptions, presented in Figure 2E, there is a significant decline up until the fifth quarter but no effect thereafter.

The results so far show the total difference between the treated and untreated individuals. To try to distinguish the origin of the effects, we present effect estimates “during” and “after” treatment in Table 6. The strategy of defining the “during” and, hence implicitly, “after” was given in the Empirical Strategy section when describing the differences between treated and controls during the period of CBT treatments in Table 6. The first two columns show the differences during treatment. Treated individuals who were not on sick leave at the initiation of treatment had 0.66 fewer days on sick leave, 3.9 more health care visits, and similar levels of drug prescriptions as the untreated matches. Treated individuals who were on sick leave had 8.8 days more of sick leave during the treatment period than the untreated matches. Similar to those not on sick leave, however, the treated individuals who were on sick leave had four more health care visits and similar levels of drug prescriptions as the untreated matches.

The last two columns of Table 6 show decreases in all variables for both groups of treated compared to their untreated matches after treatment, although the decrease in sick leave days for those on sick leave is not statistically significant. For those not on sick leave, sick days decreased by 6.1 days, the number of health care visits decreased by 3.2, and the number of drug prescriptions decreased by 1.4 after treatment compared to the untreated matches. For the treated on sick leave, the number of health care visits decreased by 4 and the number of drug prescriptions decreased by 2.1 compared to the untreated matches during the period after treatment. Overall, the results in Table 6 support that there are positive effects of the CBT treatment, which primarily show up after the treatment period.

### Sensitivity Analysis

As discussed above, there is a risk that the matching estimator is biased as it does not account for all factors that are jointly important for the treatment and the outcome if not treated. In this particular case, the assessment of the prescribing doctor could, for example, be based on information that cannot be observed in the data. The fact that there is no difference in pretreatment sick leave provides some support that the bias may not be large. An additional support is that expected effects on the number of health care visits and drug prescriptions, that is, outcome measures that should be directly related to treatment, are found in the short run. If the selection to treatment within the MRG would be based on unobservables, effects should have appeared gradually and not immediately (see, e.g., Abbring & van den Berg, 2003).

Even if these two results speak against unobservable factors having skewed our results, a more formal test can be pursued using a variable not included in the set of matching variables. The maintained assumption is that this variable is not related to the outcome given the set of matching covariates under the null of no confounding. Here, we use the number of contracted clinics to provide CBT within the MRG in the area of residence of the individual (see Figures A1 and A2 for a description of the variation over time and across areas). The argument for this variable to be valid is that the governmental initiative changed the number of contracted clinics exogenously. That is, we assume that given the set of covariates, the number of contracted clinics is not driven by demand (i.e., health and preferences).

The intuition behind the test suggested in de Luna and Johansson (2014) is straightforward. If we divide the sample into high and low number of clinics in the area of residence at a specific time, the means of the outcome if not obtaining CBT in areas or time periods with many or few clinics should be the same if there is no confounding.

In order for the test to be relevant (have power), the instrument needs to affect the assignment to treatment. Let an individual in the matched population be given the value 1 if the number of contracted clinics in the surrounding area is above the median number, otherwise 0. Regressing this discretized instrument on CBT for the matched sample, we find that the probability of receiving CBT increases by 1.2 (*F* value = 234.57) and 1.0 (*F* value = 92.48) percentage points for individuals on sick leave and not on sick leave at the start of treatment, respectively (see Columns 1 and 3in Table A4).

The resulting test shows that there are no differences in sick leave for individuals not given CBT in areas/time periods when the number of clinics is above the median than in areas/time periods when the number of clinics is below the median (see the test given in Columns 2 and 4 in Table A4). It is hence not likely that the results stemming from the matching estimator are due to confounding.

## Public Finance Implications

Given the above results, a natural question is whether CBT is justified from a public finance perspective. Table 7 presents rough calculations of the public costs and benefits of the CBT during the follow-up period of up to 2 years after the start of treatment.

**Table 7.** Public Finance Implications, SEK.

Variables	Sick Leave Payments	Outpatient Care Visits	Subsidized Value of Drug Prescriptions	Public Finance Implications, Total
Not on sick leave	−1,748	2,084	−341	−5
On sick leave	1,136	1,706	509	3,351

*Note.* The outcome variables are measured during the year following the initiation of treatment. The public finance implications are the sum of sick leave payments, the outpatient care visits with mental illness diagnosis times an estimated cost per visit of SEK 1,200, and the subsidized value of drug prescriptions. Number of observations, not on sick leave: 17,561, on sick leave: 4,240.

The analysis accounts for changes in sick leave payments (including sickness and disability benefits), health care visits with a mental illness diagnosis, and drug prescriptions. The sick leave costs are estimated directly using data on benefits payments. For health care visits, we use the estimates for the effect on the number of visits, presented in Table 7, and assign a cost per visit of SEK 1,200, which is the average cost for different types of visits. Also, the costs of drug prescriptions can be estimated directly using the subsidized value of drug prescriptions above what the individual pays out of their pocket.

The public finance calculations should be interpreted with caution. First, the previous analysis suggested that some of the effects may last for more than 2 years, and a longer follow-up period may therefore give different results. Second, there may be public costs and benefits that are not included in the calculations. For example, since sickness benefits do not fully compensate for income loss, tax revenues would increase with reduced work absence. This is partly due to increased income taxes but also due to increased value added taxes if the additional income is used for consumption. Third, potential benefits in terms of increased well-being are also not considered. Finally, the productivity at the workplace may be affected by the treatment even if it does not spill over into sick leave. For all these reasons, the public finance calculations should merely be seen as an indication of the potential impact on public finances.

The first column of Table 7 shows a reduced cost for sick leave payments of about SEK 1,700 per patient for those not on sick leave when treatment was initiated and an increased cost of about SEK 1,100 per individual for those listed sick at the time of treatment. The second column shows an increase in the number of health care visits, valued at around SEK 2,000 for those not on sick leave and SEK 1,700 for those on sick leave. The third column shows a decrease by SEK 341 in the subsidized value of drug prescriptions for those not sick listed at the time of treatment and an increase by SEK 509 for those sick listed at the time of treatment. Finally, the fourth column shows the total costs and savings. The positive effects from CBT for individuals not on sick leave at the initiation of treatment were large enough to compensate for the costs of more health care visits during the treatment period. This was not the case for the individuals already on sick leave.

## Conclusion

Labor market exclusion due to mental illness is a key concern for policy makers around the world. Despite the large number of individuals who suffer from mental illness, the evidence on the effectiveness of different types of treatment is still scarce. In particular, we know little about the impact on employment outcomes. We also know little about when during the course of the disease different types of treatment are most effective.

In this article, we have studied the impact of CBT for individuals with mild or moderate mental illness. We utilized a government initiative providing additional funding for CBT, which increased the supply of treatment. To study the impact of CBT at different stages of the sickness episode, the analysis was performed separately for individuals who were not on sick leave and individuals who were on sick leave when treatment was initiated.

The results suggest that CBT reduced sick leave and drug prescriptions for individuals who were not on sick leave at the initiation of treatment. It initially increased the number of health care visits with a mental illness diagnosis, but this effect was reversed to a reduced number of visits in the long run. For individuals who were on sick leave, we find no reduction in sick leave and no long-term decrease in health care visits or drug prescriptions. This, thus, suggests that CBT is most effective at an early stage of an illness as a preventive measure rather than a measure to promote return to work.

Previous research has shown that sick leave is affected by many factors other than the working capacity of the individual, for example, the benefit level (see, e.g., Henrekson & Persson, 2004; Johansson & Palme, 1996, 2002, 2005; Larsson, 2006; Skogman Thoursie, 2004) and screening stringency (see, e.g., Hartman et al., 2013; Johansson & Lindahl, 2013). Studies have also shown that rehabilitation measures may increase rather than decrease sick leave by giving the individual an identity as sick or by creating lock-in effects, that is, preventing work during the rehabilitation period. This may imply that it is more difficult to achieve positive results for individuals who are already on sick leave compared to those who are still working. In a survey of vocational rehabilitation, Johansson et al. (2011) conclude that measures at the workplace appear to be most effective. Our results also indicate that the connection to the work place may be an important dimension.

An interesting question for further research is the role of treatment at different stages of the sickness episode. The positive effects of CBT for individuals not on sick leave were a result of the alternative treatment, apart from medication, being sick leave. To not put individuals on sick leave during the CBT had positive effects in the form of lower sick leave during and after treatment. To combine rehabilitation with work appears to be a good idea to achieve positive results.

## Figures and Tables

**Figure A5. fig7-0193841X20976516:**
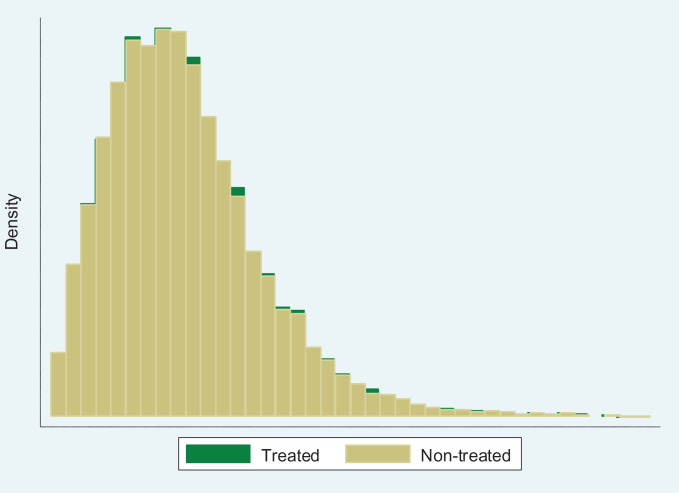
Distribution of estimated propensity scores for the treated and nontreated for the cognitive behavioral therapy sample after matching, not on sick leave.

**Figure A6. fig8-0193841X20976516:**
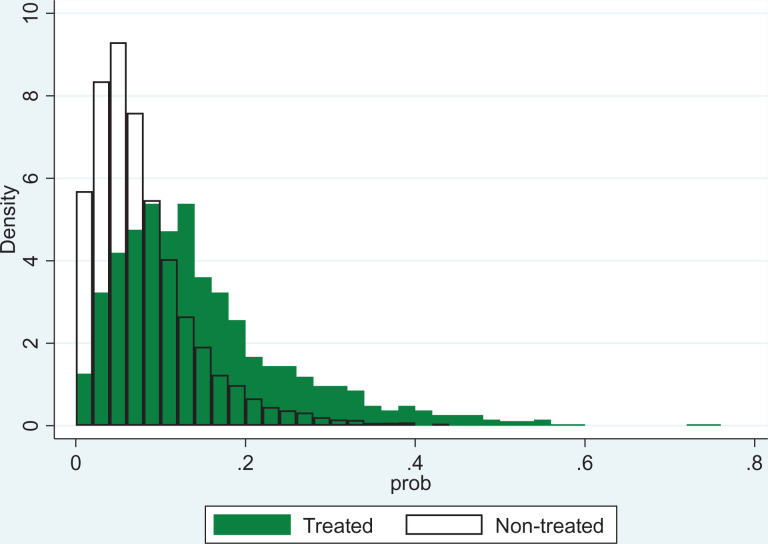
Distribution of estimated propensity scores for the treated and nontreated for the cognitive behavioral therapy sample before matching, on sick leave.

**Figure A7.**
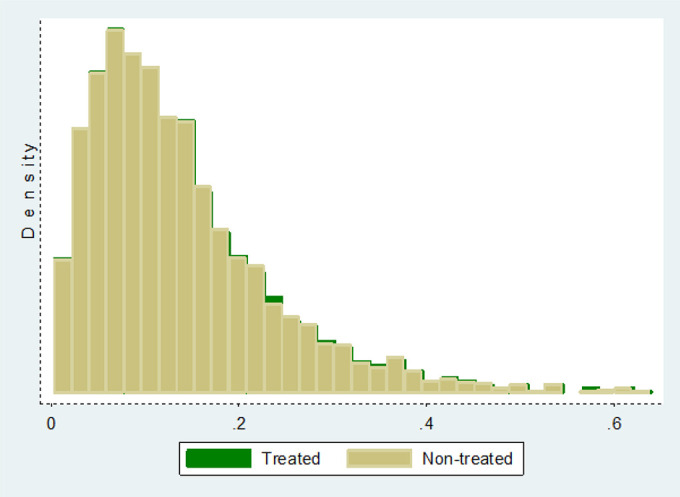
Distribution of estimated propensity scores for the treated and nontreated for the cognitive behavioral therapy sample after matching, on sick leave.

**Figure A8.**
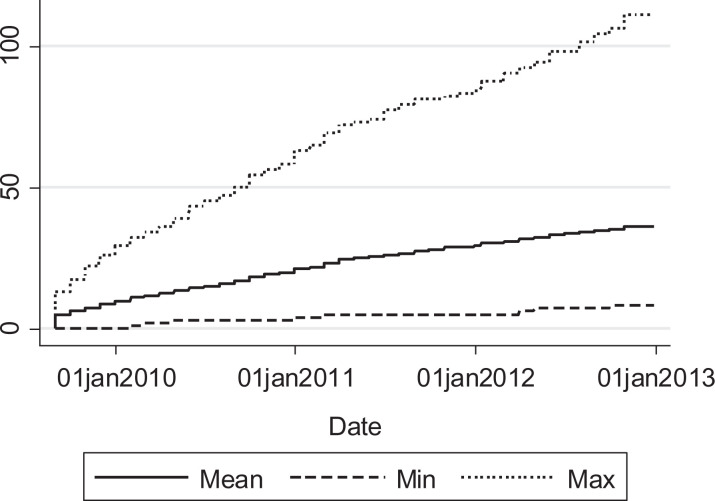
Mean, minimum, and maximum of the instrument (the number of clinics with a contract to perform cognitive behavioral therapy within the medical rehabilitation guarantee in the own municipality and surrounding municipalities combined) across the Skåne municipalities from September 1, 2009, to December 31, 2012.

## Appendix A

**Table A1.** Diagnoses Covered by the Medical Rehabilitation Guarantee.

F32	Depressive episode
F33	Recurrent depressive disorder
F40	Phobic anxiety disorders
F41.0	Panic disorder
F41.1	Generalized anxiety disorder
F41.9	Anxiety disorder, unspecified
F42	Obsessive-compulsive disorder
F43.1	Post-traumatic stress disorder
F43.8	Other reactions to severe stress
F43.9	Reaction to severe stress, unspecified

**Table A2.** Estimation of the Propensity to Receive Cognitive Behavioral Therapy, Not on Sick Leave.

Variables/parameters	Estimate	Standard Error	*p* Value
Intercept	−2.299	.071	<.0001
Year 2012	0.324	.023	<.0001
Male	−0.186	.024	<.0001
Married	−0.114	.025	<.0001
Age	−0.011	.001	<.0001
Foreign born	−0.471	.033	<.0001
Earnings	0.000	.000	<.0001
Education			
High school	0.560	.042	<.0001
College	0.946	.043	<.0001
Missing	−0.824	.147	<.0001
Unemployed	−0.116	.030	.0001
Labor market status in November last year			
Not employed, with earnings statement	−0.095	.032	.003
Not employed, without earnings statement	−0.211	.042	<.0001
Outpatient care visits			
Total, since 2008	0.097	.013	<.0001
Total, Quarter 1	−0.067	.004	<.0001
Total, Quarter 2	−0.007	.004	.060
Doctor visits, Quarter 1	0.446	.011	<.0001
Doctor visits, Quarter 2	0.291	.012	<.0001
Doctor visits, Quarter 3	0.117	.012	<.0001
Doctor visits, Quarter 4	−0.040	.014	.005
Mental illness diagnosis, since 2008	−0.209	.007	<.0001
Mental illness diagnosis, Quarter 4	−0.144	.038	.0001
Pain-related diagnosis, since 2008	−0.070	.010	<.0001
Pain-related diagnosis, Quarter 2	0.168	.028	<.0001
Primary care, since 2008	−0.095	.013	<.0001
Specialist care, since 2008	−0.093	.013	<.0001
Specialist care, Quarter 1	−0.378	.018	<.0001
Specialist care, Quarter 2	−0.123	.017	<.0001
Specialist care, Quarter 4	0.065	.016	<.0001
Drug prescriptions			
Number of prescriptions since 2008	−0.003	.000	<.0001
Number of prescriptions, Quarter 1	0.015	.003	<.0001
Value of prescriptions, during last year	0.000	.000	.062

*Note*. In the estimation, we also include diagnosis code (10 categories) and calendar month of the health care visit (12 categories).

**Table A3.** Estimation of the Propensity to Receive Cognitive Behavioral Therapy, on Sick Leave.

Variables/parameters	Estimate	Standard Error	*p* Value
Intercept	−3.157	.146	<.0001
Year 2012	−0.331	.044	<.0001
Male	−0.149	.051	.004
Age	−0.010	.002	<.0001
Foreign born	−0.370	.065	<.0001
Earnings (SEK 1,000)	0.000	.000	.000
Education			
High school	0.282	.080	.000
College	0.574	.082	<.0001
Missing	−0.322	.528	.542
Unemployed	−0.078	.066	.244
Outpatient care visits			
Total, Quarter 1	−0.056	.005	<.0001
Doctor visits, Quarter 1	0.290	.015	<.0001
Doctor visits, Quarter 2	0.075	.017	<.0001
Mental illness diagnosis, since 2008	−0.203	.023	<.0001
Mental illness diagnosis, during last year	0.365	.040	<.0001
Mental illness diagnosis, Quarter 2	−0.480	.048	<.0001
Mental illness diagnosis, Quarter 3	−0.329	.059	<.0001
Mental illness diagnosis, Quarter 4	−0.452	.073	<.0001
Specialist care, Quarter 1	−0.151	.018	<.0001
Number of drug prescriptions, Quarter 1	0.015	.004	<.0001
Sickness benefit days			
Days, Quarter 1	0.025	.001	<.0001
Days, Quarter 2	−0.004	.001	.000
Days during last 3 years	−0.000	.000	.010
Full-time sick leave	−0.245	.061	<.0001
Outpatient care visits, mental illness diagnosis, Quarter 1* Number of sickness benefit days, Quarter 1	−0.008	.001	<.0001

*Note*. In the estimation, we also included diagnosis (10 categories).

**Table A4.** Results From Test of Matching Strategy, Cognitive Behavioral Therapy.

Variables/parameters	Not on Sick Leave		On Sick Leave	
	1	2	3	4
	Effect of Number of Organizers	Chow Test	Effect of Number of Organizers	Chow Test
Estimate (standard error)	0.010 (.001)		0.012 (.001)	
*F* and χ^2^ test (*p* value)	234.57 (.00)	1.87 (.17)	92.48 (.00)	0.99 (.32)

## Notes

1. Since the supply of cognitive behavioral therapy (CBT) expanded gradually, few patients received CBT in the fall 2009, and the exclusion of these patients does not affect the results.

2. The sample is a combination of the samples used in Hägglund et al. (2012, 2014). Therefore, there is a gap in the sampling of individuals during the second half of 2011.

3. In addition to the matching approach, we have tried an instrumental variable (IV) approach in which we used the number of contracted clinics to provide CBT within the medical rehabilitation guarantee in the area of residence as an instrument for individual treatment. The identifying assumption is that the distribution of potential outcomes and potential treatment assignments are independent of the instrument. We argue that this is the case since the governmental initiative is an exogenous shift in supply that did affect exposure and does not appear to be related to the local demand for CBT. The IV results turned out to be too imprecise to be informative.

4. We also, as in all evaluations at the individual level, assume that we have no interference between individuals.

5. The results are robust to different matching algorithms.

6. The same health care visit is allowed to be selected as comparison for several of the treated. In most instances, a health care visit is equivalent to a patient, but using health care visit instead of patient as the observation entity increases the possibilities of finding a relevant comparison group. A treated individual is, however, not allowed to be matched to themself before treatment.

7. Note that standard statistical *t* tests would be too restrictive, as five of 100 covariates would be statistically different by chance.

8. Among the variables in Table 3, inpatient care days and doctor visits are also not included for any group, and specialist and primary care visits are not included for those on sick leave.

9. The standard errors are estimated nonparametrically using the nearest neighbor matching estimators of Abadie and Imbens (2006).

10. Comparing impact estimates in the two periods using the same follow-up period (8 months), the differences are throughout nonsignificant and of small size. For instance, comparing sick leave days for those not on sick leave at treatment start, the impact estimate is −3.1 for those starting treatment before 2012 and −3.2 for those starting in 2012. Comparing health care visits, the impact estimates are 2.3 and 2.2 visits, respectively.
